# Acute Exfoliative Dermatitis/Erythroderma Secondary to Gliclazide

**DOI:** 10.7759/cureus.45965

**Published:** 2023-09-25

**Authors:** Saifaldeen Al-Badawi, Nada Ahmed, Mohammed Akber

**Affiliations:** 1 Internal Medicine, Birmingham Heartlands Hospital, Birmingham, GBR; 2 Dermatology, Lincoln County Hospital, Lincoln, GBR; 3 Diabetes and Endocrinology, Queen Elizabeth Hospital Birmingham, Birmingham, GBR

**Keywords:** exfoliative, dermatitis, skin rash, gliclazide, erythroderma

## Abstract

Erythroderma is a general term used to describe severe, intense skin inflammation. The condition is also known as exfoliative dermatitis when it is associated with exfoliation. Erythroderma has many causes, such as adverse drug eruption, dermatitis, psoriasis, pityriasis rubra pilaris, immunobullous disease, cutaneous T-cell lymphoma (Sézary syndrome), underlying systemic malignancy, graft versus host disease, and HIV infection. Many medications can cause erythroderma, including antibiotics, antiepileptics, angiotensin-converting enzyme (ACE) inhibitors, and sulfonamides. Here, we report a rare case of erythroderma secondary to gliclazide, an oral antidiabetic. This presentation is rare, as we found only one case report of gliclazide causing erythroderma in the literature. Erythroderma is considered a medical emergency requiring immediate diagnosis and prompt management; therefore, early intervention should start on suspicion without waiting for dermatologist confirmation, as this will significantly reduce the mortality and morbidity of this potentially life-threatening emergency.

## Introduction

Erythroderma is defined as a generalised or nearly generalised sustained erythema of the skin involving more than 90% of the body surface area with a variable degree of scaling. Some cases are also associated with erosions, crusting, and hair and nail changes. Exfoliative dermatitis and erythroderma (the preferred term) have been used synonymously in the literature [1].

Common causes of erythroderma are: Idiopathic - 30%, Infections (HIV, herpes simplex virus (HSV), dermatophytosis, scabies), Drug allergy - 20%, Seborrheic dermatitis - 2%, Sarcoidosis, Contact dermatitis - 3%, Connective tissue diseases, Atopic dermatitis - 10%, Autoimmune (systemic lupus/dermatomyositis/bullous pemphigoid/pemphigus foliaceus/lichen planus/graft vs host disease), Lymphoma and leukemia - 14% (including Sezary syndrome), Psoriasis - 23% (including reactive arthritis/pityriasis rubra pilaris) [2]. The above-mentioned examples indicate the variety of diseases that erythroderma can occur with. Erythroderma can be acute or chronic. In acute erythroderma, skin failure can lead to life-threatening systemic upset, requiring intensive care unit treatment. In chronic erythroderma, systemic complications are less frequent [3].

The clinical presentations of patients with erythroderma can vary from mild, stable case to life-threatening condition. Therefore, healthcare workers in different medical specialties and clinical settings must have sufficient knowledge of how to treat erythroderma.

Sometimes the underlying clinical condition necessitates the intervention of wound care professionals, and the dermatological team’s early involvement is vital whenever possible.

## Case presentation

Our case is a 72-year-old woman with a medical history of poorly controlled type 2 diabetes mellitus, asthma, rheumatoid arthritis, and hypothyroidism. Her regular medications include: empagliflozin, fexofenadine, etanercept, aspirin, atorvastatin, bendroflumethiazide, lansoprazole, levothyroxine sodium, metformin, and fostair and salbutamol inhalers. She presented to the emergency department with a widespread skin rash all over her body beginning two weeks ago, only sparing her face. The rash initially appeared on the abdomen and then spread to her lower limbs, arms, and back; the rash was evolving from small spots into bigger, itchy painful lesions. The rash appeared one day after starting gliclazide, prescribed by her GP. She received antihistamine and antifungal medications with no signs of improvement. She denied chest pain, shortness of breath, headaches, or fever.

**Figure 1:**
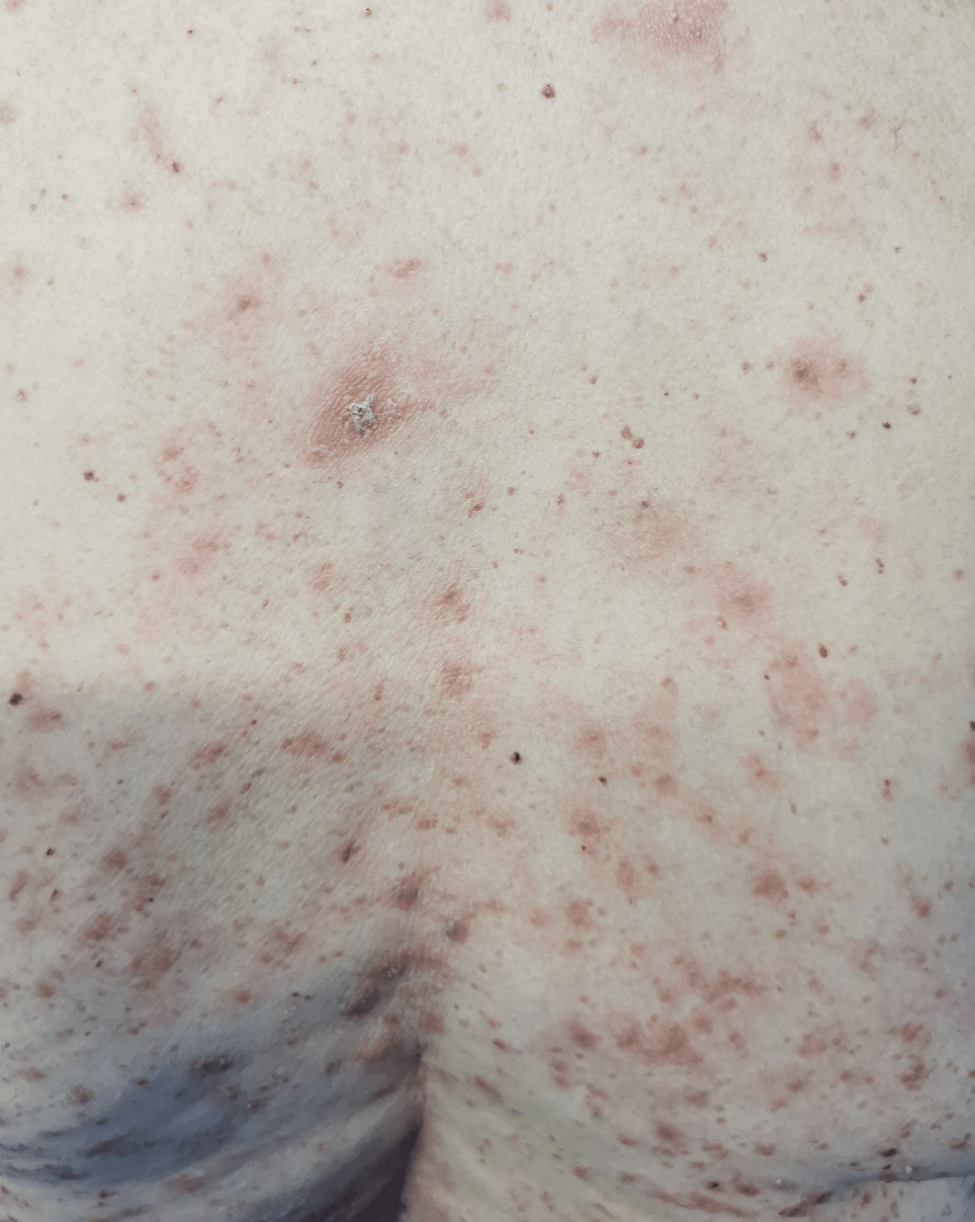
Erythrodermic rash on the back

**Figure 2:**
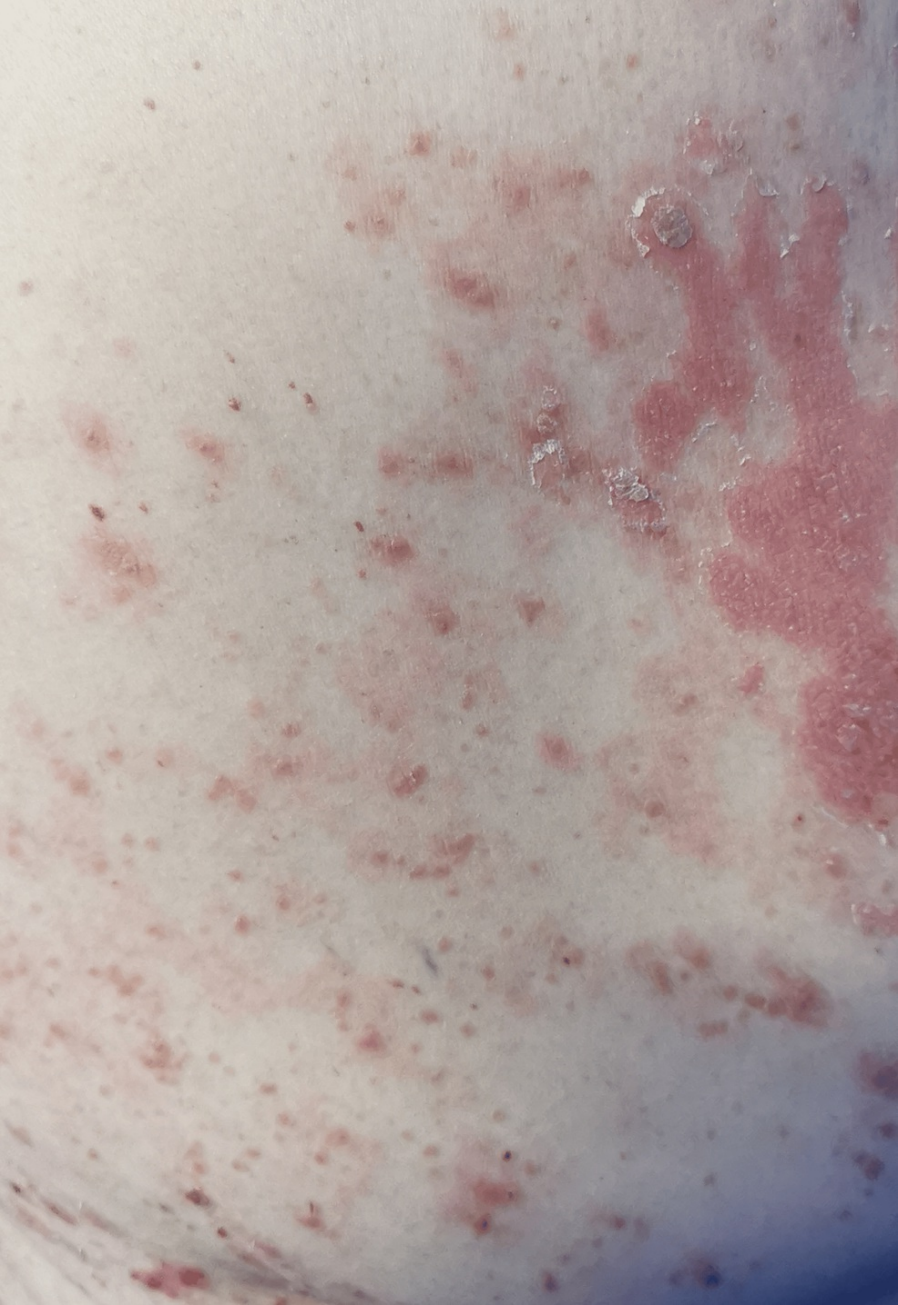
Erythrodermic rash on the right flank

**Figure 3:**
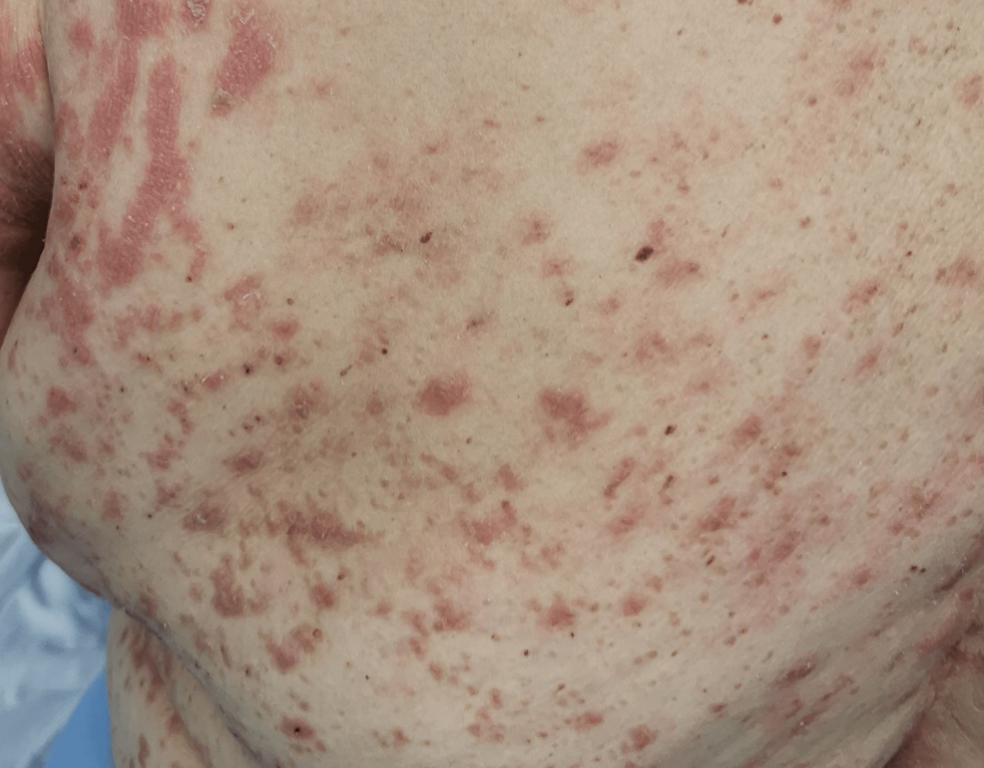
Erythrodermic rash on the back

**Figure 4:**
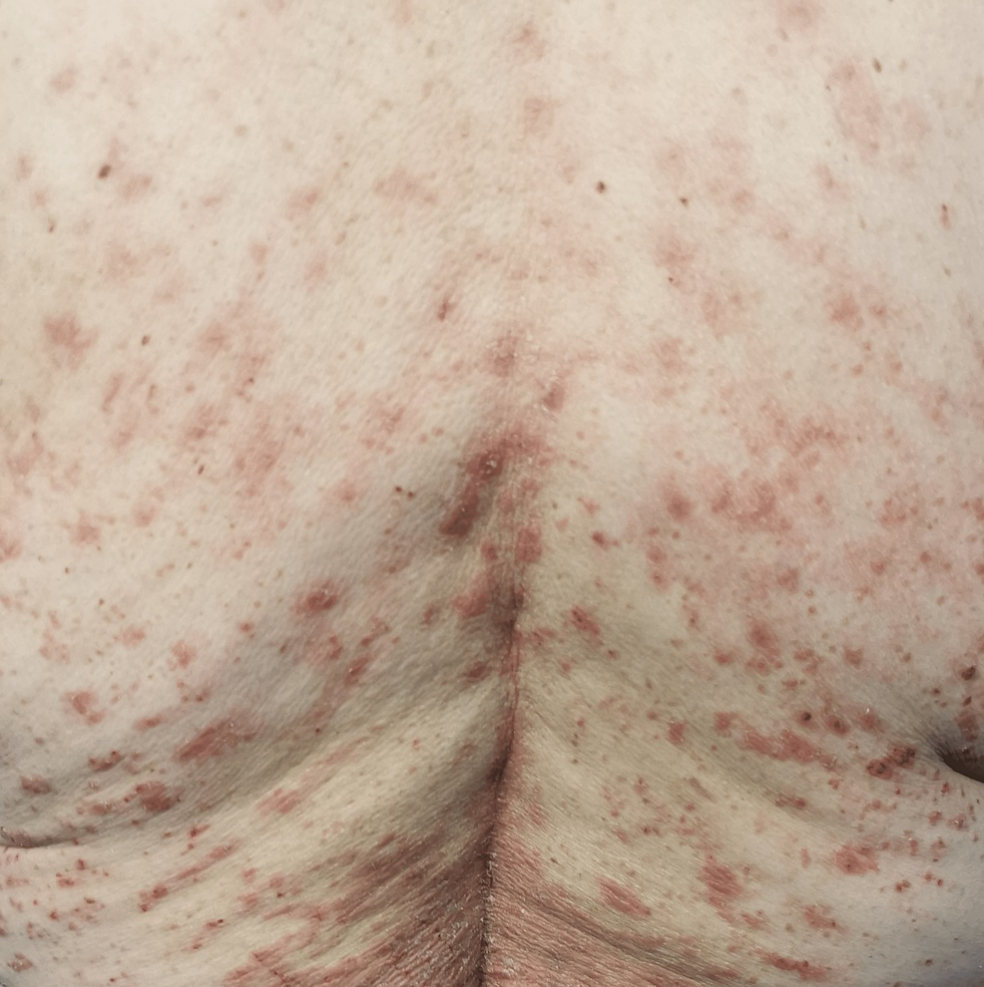
Erythrodermic rash on the back

**Figure 5:**
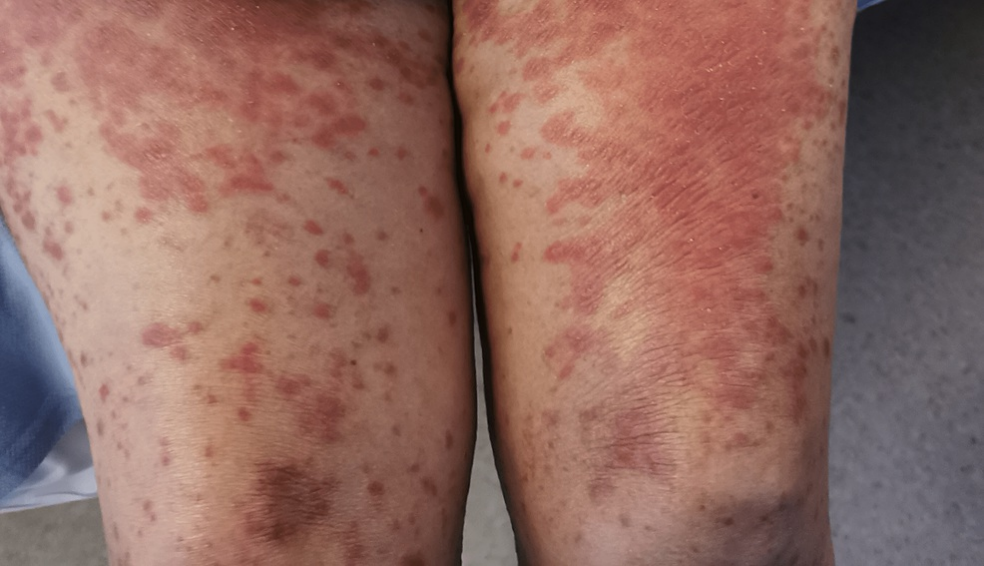
Erythrodermic rash on the thighs

**Figure 6:**
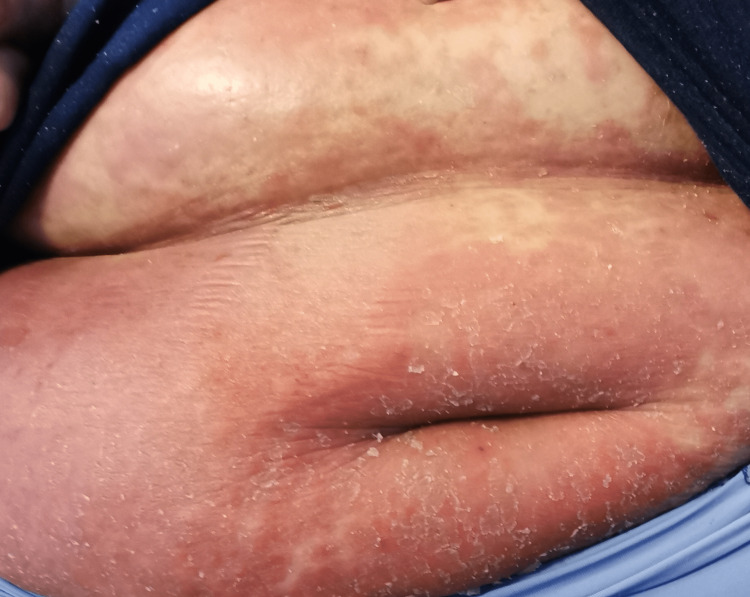
Erythrodermic rash on the abdomen

**Figure 7:**
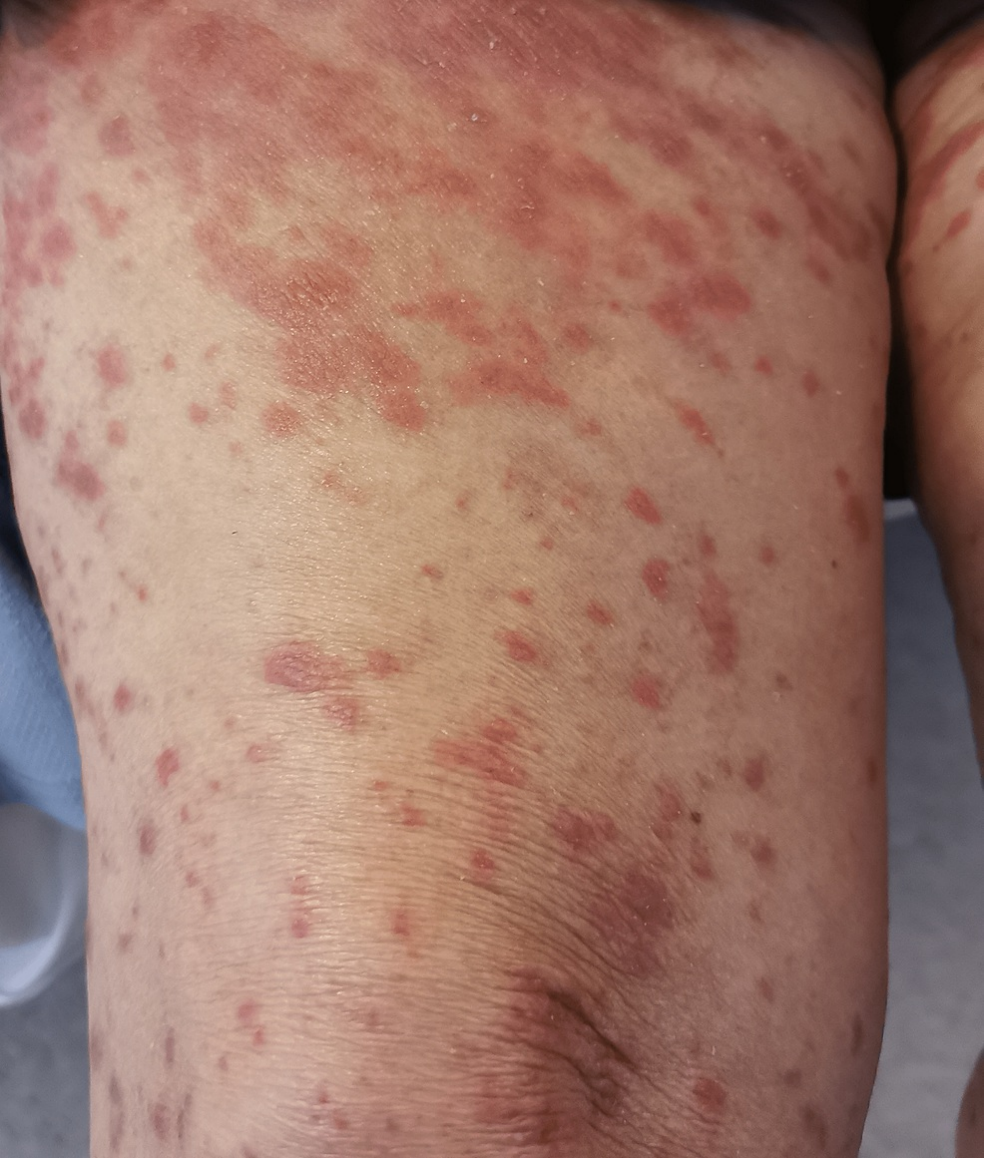
Erythrodermic rash on the right thigh

**Figure 8:**
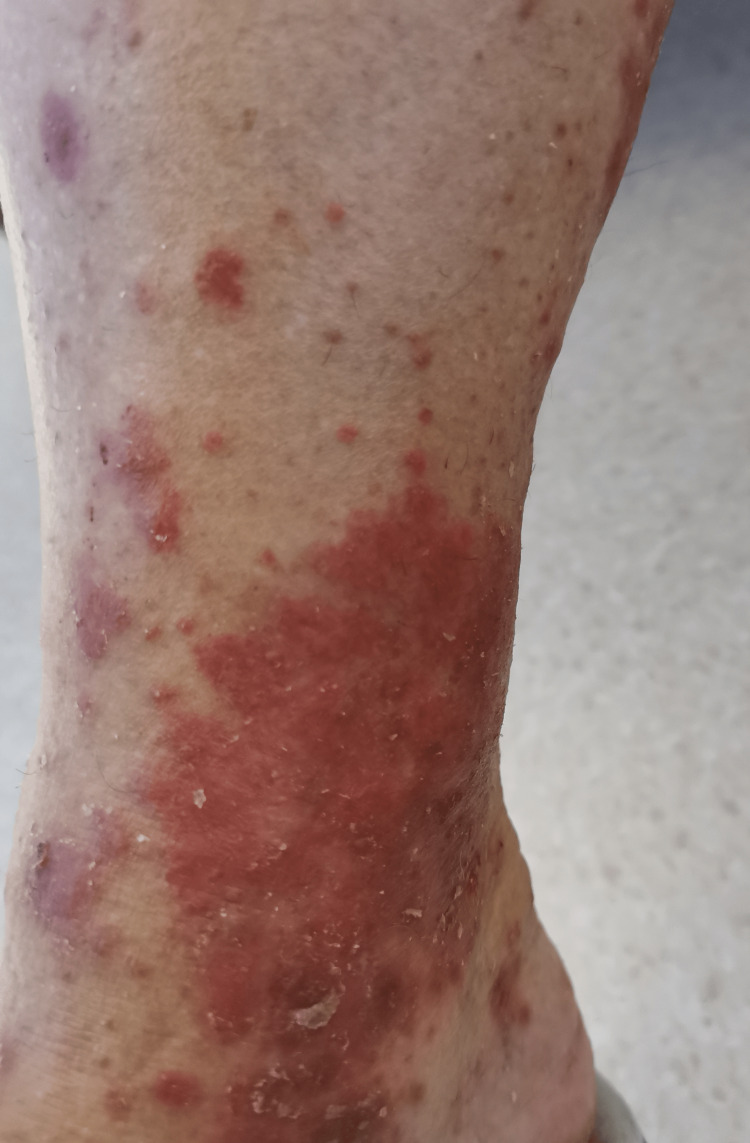
Erythrodermic rash on the lower part of the right leg

**Figure 9:**
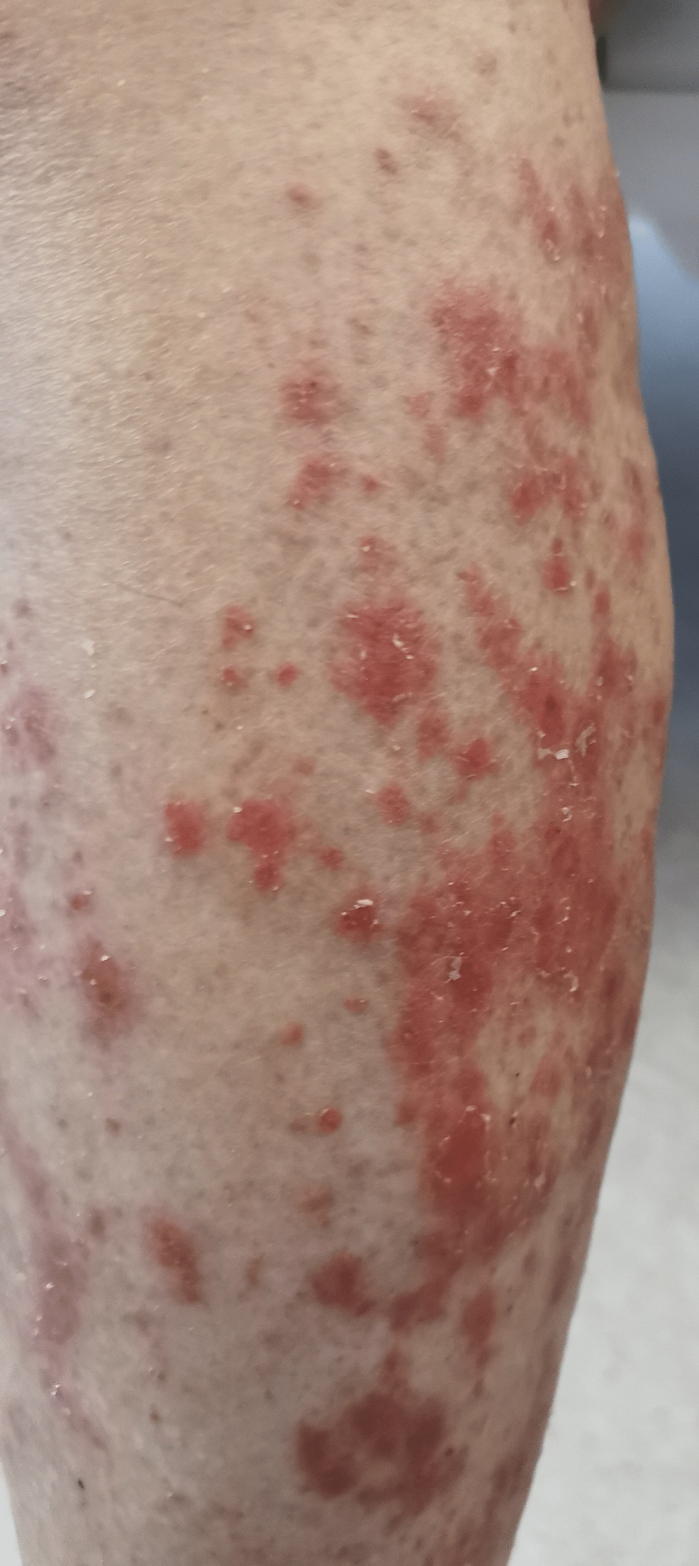
Erythrodermic rash on the middle part of the right leg

**Figure 10:**
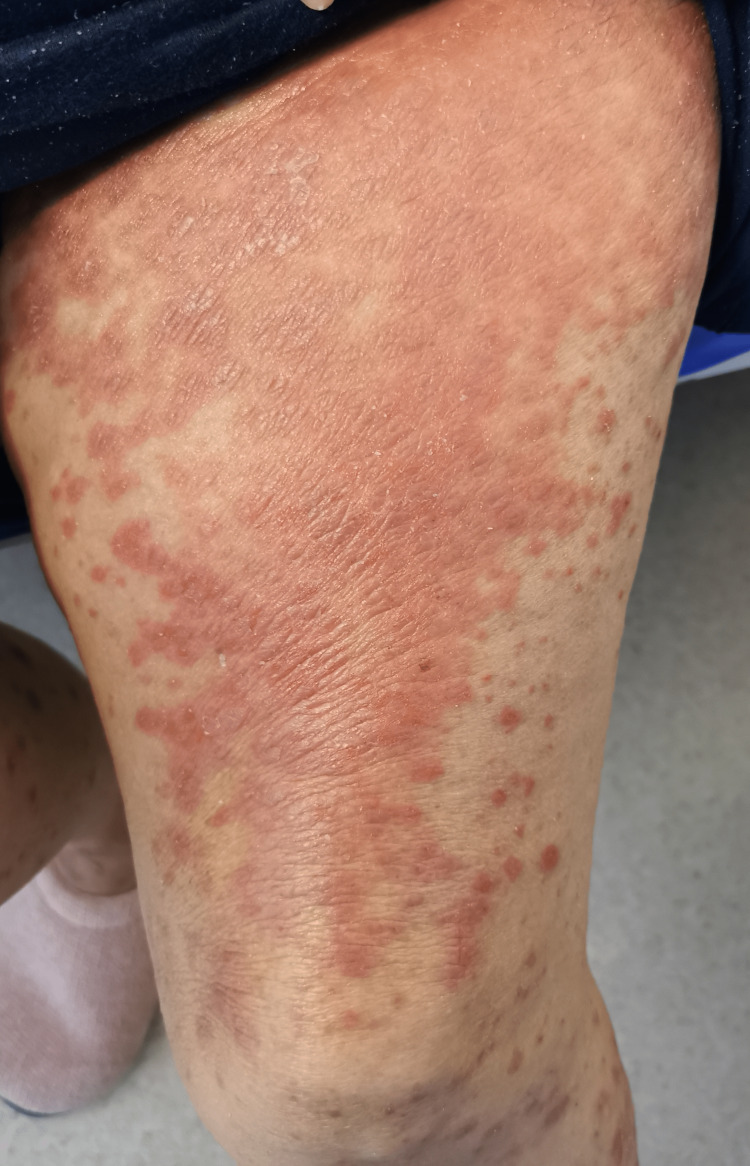
Erythrodermic rash on the left thigh

**Figure 11:**
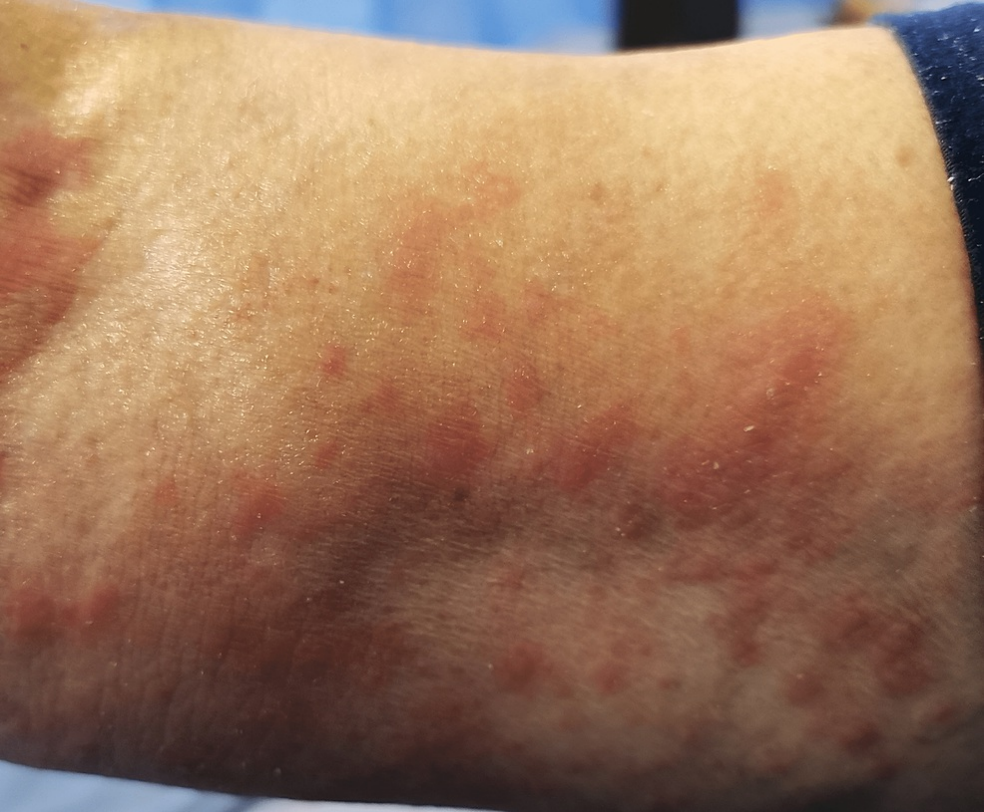
Erythrodermic rash on the right arm

**Figure 12:**
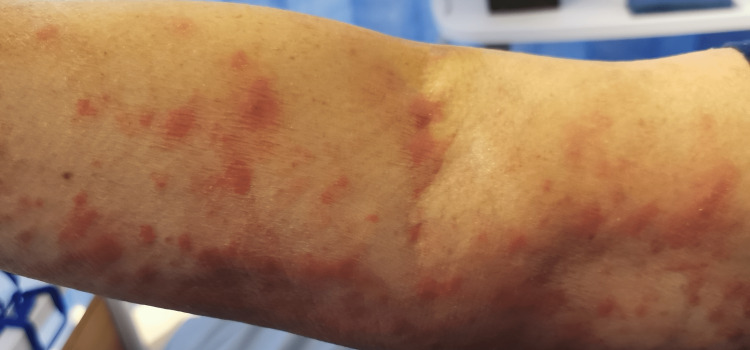
Erythrodermic rash on the right arm and forearm

In the emergency department, she was found to be afebrile, having a blood pressure of 118/81. Her initial examination revealed maculopapular, erythematous, scaly lesions involving the trunk, chest, back, forearms, dorsum of the hands, and lower limbs including palms and soles. The lesions were tender and warm, with excoriation marks. No mucosal lesions were detected. Otherwise, her systemic examination was unremarkable. Figures 1-12 show erythrodermic rash on different parts of her body.

Her labs showed: C-reactive protein (CRP) 16 (elevated) (standard 0-5 mg/L); WBCs 17.37 (elevated) (standard 3.00-10.90 ×10*9/L); neutrophils 10.93 (elevated) (standard 1.50- 7.10 ×10*9/L); eosinophils 0.33 (elevated) (standard 0.03-0.28 ×10*9/L). Urea and electrolytes, liver function tests (LFTs), and bone profile were within normal limits. Blood glucose was 13.9 (elevated) (standard 3.9-5.8 mmol/L). Immunological screening was done to exclude autoimmune diseases: rheumatic factor 73.2 (elevated) (standard 0.0-29.9 IU/ml). Liver/kidney microsome, anti-smooth muscle, and anti-mitochondrial antibodies were negative. C3 was 1.54 (normal) (standard 0.75-1.65 g/L), and C4 was 0.20 (normal) (standard 0.14- 0.54 g/L). Her chest X-ray was unremarkable. The patient was admitted to the medical assessment unit, and gliclazide was immediately stopped while insulin glargine (Lantus; Sanofi-Aventis, Paris, France)** **16 units OD was commenced. Naranjo scale was 7 points, which means probable adverse drug reaction. After review by the dermatology team a diagnosis of drug-induced acute exfoliative dermatitis/erythroderma secondary to gliclazide was suspected, they started her on Dermol 500 lotion (Benzalkonium chloride and chlorhexidine dihydrochloride + Liquid paraffin and isopropyl myristate; Dermal Laboratories Ltd., Hitchin, UK) as a soap substitute, 50/50 white soft/liquid paraffin as an emollient four times a day, and betamethasone valerate 0.025% ointment twice a day to all red areas for five days then switching to once a day. Although her skin lesions were extensive, topical steroids were preferred over oral steroids to avoid further worsening of her glycaemic control and because she remained clinically stable otherwise.

Although her urinalysis showed positive nitrate, we decided to withhold antibiotics considering the skin rash, absence of symptoms of UTI, and negative urine culture. The patient's rash showed improvement with gradual disappearance from her palms and soles since the second day of starting the treatment. The dermatology team decided that a biopsy was not needed as her skin rash responded well to the treatment. On further monitoring of her bloods after two days, WBCs decreased to 10.30 (standard 3.00-10.90 ×10*9/L), neutrophils decreased to 5.59 (standard 1.50- 7.10 ×10*9/L), and eosinophils increased to 0.47 (standard 0.03-0.28 ×10*9/L).

She remained inpatient for a few days and continued to show improvement day after day. The dermatology team reviewed her again before discharge and planned for betamethasone valerate to continue twice a day for two weeks, then on alternate days for another two weeks, and then twice weekly for two weeks.

She was discharged with a plan in place for a follow-up at the outpatient dermatology clinic which revealed complete resolution of her skin lesions after six weeks and she was advised to continue using Dermol 500 lotion and 50/50 white soft/liquid paraffin indefinitely.

## Discussion

Erythroderma is the term used to describe intense and usually widespread reddening of the skin, also known as exfoliative dermatitis. Erythroderma is rare but can arise at any age and in people of all races. It is about three times more common in males than in females. Most people have a preexisting skin disease or a systemic condition known to be associated with erythroderma. About 30% of erythroderma cases are idiopathic [4]. One of the most common causes is drug hypersensitivity reactions.

Gliclazide is an oral antihyperglycemic agent used for the treatment of non-insulin-dependent diabetes mellitus (NIDDM). It belongs to the sulfonylurea class of insulin secretagogues, which act by stimulating β cells of the pancreas to release insulin. Gliclazide binds to the β cell sulfonylurea receptor (SUR1). This binding subsequently blocks the adenosine triphosphate (ATP)-sensitive potassium channels. The binding results in the closure of the channels and leads to a resulting decrease in potassium efflux, leading to depolarization of the β cells. This opens voltage-dependent calcium channels in the β cell resulting in calmodulin activation, which in turn leads to exocytosis of insulin-containing secretory granules [5]. Its most notable adverse effects are hypoglycaemia and gastrointestinal disturbances such as constipation, nausea, epigastric discomfort, and heartburn [5].

Gliclazide with related erythroderma was found in only one case report. Erythroderma is not a known side effect associated with it. Other treatments for diabetes mellitus have also been linked to erythroderma in the literature, in one study. Metformin proved to be the cause after 4 years of symptomatic erythroderma [6].

The pathophysiologic processes resulting in exfoliative dermatitis vary with the underlying disorder responsible for the dermatitis. However, common to all conditions that cause exfoliative dermatitis is an increased rate of skin turnover. The normal epidermis has a continual turnover of epithelial cells. Cell division occurs near the basal layer. As cells move toward the periphery, they become well-keratinized. This process requires approximately 10-12 days. Cells subsequently remain in the stratum corneum for another 12-14 days prior to being sloughed [7]. The Naranjo scale was 7 points as mentioned in the case presentation, which means probable adverse drug reaction. The Naranjo scale, or Naranjo Nomogram is a questionnaire designed by Naranjo et al. for determining the likelihood of whether an adverse drug reaction is actually due to the drug rather than the result of other factors. Probability is assigned via a score termed definite, probable, possible, or doubtful [8].

We reviewed her other drug history and found no interactions with glicalazide. The impression was acute exfoliative dermatitis/erythroderma, and the cause proved to be secondary to Gliclazide. We reviewed this case with the history provided and the findings in the examination; the dermatology team was involved, and the diagnosis was proven mainly clinically.

The generalizability of the results made the confirmation difficult, but with the absence of other causes, erythroderma was the only obvious reason for this presentation.

## Conclusions

We concluded from this case report that gliclazide can cause erythroderma. Although in the literature review, this is a rare side effect among other skin manifestations related to gliclazide and among causes of erythroderma, these results should be considered when prescribing gliclazide in the future. Further studies will be beneficial in the future to highlight this side effect, its incidence, and if it is related to any other triggering factors. Treatment of erythroderma can be challenging sometimes, but identifying the cause is of great importance to optimize the management plan.
